# Supplementary material for: Modeling glioblastoma heterogeneity as a dynamic network of cell states
Source: Mol Syst Biol. 2021 Sep 16;17(9):e10105. doi: 10.15252/msb.202010105 (PMC8444284; doi:10.15252/msb.202010105)
Supplement: Supplementary file 6 — Source Data for Figure 5 [file MSB-17-e10105-s004.zip › Figure5A_sourcedata/GSEA_3071/nosigdb_stateV.GseaPreranked.1622795159954/gsea_report_for_na_neg_1622795159954.html]

Report for na\_neg 1622795159954 [GSEA]

| GS  follow link to MSigDB | GS DETAILS | SIZE | ES | NES | NOM p-val | FDR q-val | FWER p-val | RANK AT MAX | LEADING EDGE || 1 | OLSEN\_MESENCHYMAL | Details ... | 72 | -0.75 | -3.86 | 0.000 | 0.000 | 0.000 | 513 | tags=63%, list=10%, signal=68% |
| 2 | IVY\_CTMVP | Details ... | 84 | -0.71 | -3.82 | 0.000 | 0.000 | 0.000 | 323 | tags=49%, list=6%, signal=51% |
| 3 | NB\_KI\_CLUSTER18 | Details ... | 70 | -0.73 | -3.77 | 0.000 | 0.000 | 0.000 | 423 | tags=59%, list=8%, signal=63% |
| 4 | DOROSZKO\_U3054MG\_CLUSTER\_2 | Details ... | 263 | -0.57 | -3.75 | 0.000 | 0.000 | 0.000 | 675 | tags=44%, list=13%, signal=48% |
| 5 | RICHARDS\_VERHAAK\_CANCERCELL\_2010\_MESENCHYMAL | Details ... | 98 | -0.65 | -3.68 | 0.000 | 0.000 | 0.000 | 746 | tags=58%, list=14%, signal=66% |
| 6 | WANG\_MESENCHYMAL\_PMID\_28697342 | Details ... | 29 | -0.83 | -3.52 | 0.000 | 0.000 | 0.000 | 479 | tags=72%, list=9%, signal=79% |
| 7 | ANASTASSIOU\_MULTICANCER\_INVASIVENESS\_PMID\_22208948 | Details ... | 42 | -0.78 | -3.51 | 0.000 | 0.000 | 0.000 | 284 | tags=55%, list=5%, signal=57% |
| 8 | DALMO\_EXTINCTION\_UNPUB | Details ... | 29 | -0.83 | -3.42 | 0.000 | 0.000 | 0.000 | 479 | tags=72%, list=9%, signal=79% |
| 9 | VANGRONINGEN\_MES | Details ... | 294 | -0.50 | -3.36 | 0.000 | 0.000 | 0.000 | 1128 | tags=53%, list=21%, signal=64% |
| 10 | RICHARDS\_NOWAKOWSKI\_SCIENCE\_2017\_MURAL\_UPREG | Details ... | 425 | -0.47 | -3.30 | 0.000 | 0.000 | 0.000 | 1038 | tags=45%, list=20%, signal=51% |
| 11 | GANDAL\_IBD\_UP\_PMID29439242 | Details ... | 64 | -0.64 | -3.30 | 0.000 | 0.000 | 0.000 | 463 | tags=45%, list=9%, signal=49% |
| 12 | SUVA\_MES1\_PMID\_31327527 | Details ... | 39 | -0.71 | -3.22 | 0.000 | 0.000 | 0.000 | 675 | tags=64%, list=13%, signal=73% |
| 13 | RICHARDS\_NEFTEL\_CELL\_2019\_MES1 | Details ... | 39 | -0.71 | -3.20 | 0.000 | 0.000 | 0.000 | 675 | tags=64%, list=13%, signal=73% |
| 14 | NB\_KI\_CLUSTER11 | Details ... | 39 | -0.70 | -3.14 | 0.000 | 0.000 | 0.000 | 794 | tags=74%, list=15%, signal=87% |
| 15 | NB\_KI\_CLUSTER1 | Details ... | 26 | -0.76 | -3.04 | 0.000 | 0.000 | 0.000 | 1186 | tags=96%, list=22%, signal=123% |
| 16 | SEGERMAN\_MULTITHERAPY\_RESISTANCE\_FIG5A\_PMID\_27974212 | Details ... | 23 | -0.75 | -2.93 | 0.000 | 0.000 | 0.000 | 1150 | tags=87%, list=22%, signal=111% |
| 17 | GANDAL\_AUTISM\_UP\_PMID29439242 | Details ... | 65 | -0.57 | -2.82 | 0.000 | 0.000 | 0.000 | 766 | tags=52%, list=14%, signal=60% |
| 18 | LARSSON\_STATE\_1 | Details ... | 69 | -0.49 | -2.56 | 0.000 | 0.000 | 0.000 | 644 | tags=46%, list=12%, signal=52% |
| 19 | NB\_KI\_CLUSTER13 | Details ... | 96 | -0.45 | -2.46 | 0.000 | 0.000 | 0.001 | 1504 | tags=64%, list=28%, signal=87% |
| 20 | NB\_KI\_CLUSTER17 | Details ... | 104 | -0.43 | -2.39 | 0.000 | 0.000 | 0.001 | 1185 | tags=54%, list=22%, signal=68% |
| 21 | SINGH\_SOX2\_DOWNREGULATED\_TRANSCRIPTS\_PMID\_28122245 |  | 74 | -0.45 | -2.36 | 0.000 | 0.000 | 0.001 | 970 | tags=45%, list=18%, signal=54% |
| 22 | RICHARDS\_LIDDELOW\_NATURE\_2017\_A2\_REACTIVE\_ASTROCYTES |  | 26 | -0.60 | -2.34 | 0.000 | 0.000 | 0.001 | 441 | tags=54%, list=8%, signal=58% |
| 23 | RICHARDS\_NOWAKOWSKI\_SCIENCE\_2017\_CHOROID\_UPREG |  | 331 | -0.34 | -2.33 | 0.000 | 0.000 | 0.001 | 1197 | tags=40%, list=23%, signal=49% |
| 24 | RICHARDS\_ZHONG\_NATURE\_2018\_MICROGLIA\_UPREG |  | 150 | -0.38 | -2.32 | 0.000 | 0.000 | 0.002 | 887 | tags=37%, list=17%, signal=43% |
| 25 | SUVA\_MES2\_PMID\_31327527 |  | 35 | -0.53 | -2.30 | 0.000 | 0.000 | 0.002 | 816 | tags=57%, list=15%, signal=67% |
| 26 | RICHARDS\_NEFTEL\_CELL\_2019\_MES2 |  | 35 | -0.53 | -2.29 | 0.000 | 0.000 | 0.002 | 816 | tags=57%, list=15%, signal=67% |
| 27 | RICHARDS\_NOWAKOWSKI\_SCIENCE\_2017\_MICROGLIA\_UPREG |  | 401 | -0.32 | -2.26 | 0.000 | 0.000 | 0.002 | 1171 | tags=40%, list=22%, signal=48% |
| 28 | IVY\_CTPAN |  | 86 | -0.41 | -2.20 | 0.000 | 0.000 | 0.006 | 964 | tags=45%, list=18%, signal=55% |
| 29 | DOROSZKO\_U3013MG\_CLUSTER\_1 |  | 28 | -0.53 | -2.18 | 0.000 | 0.000 | 0.007 | 174 | tags=29%, list=3%, signal=29% |
| 30 | NB\_KI\_CLUSTER12 |  | 39 | -0.47 | -2.10 | 0.000 | 0.001 | 0.017 | 473 | tags=31%, list=9%, signal=34% |
| 31 | NB\_KI\_CLUSTER16 |  | 69 | -0.40 | -2.06 | 0.000 | 0.001 | 0.022 | 579 | tags=33%, list=11%, signal=37% |
| 32 | OLSEN\_SCPLIKE |  | 62 | -0.40 | -2.04 | 0.000 | 0.001 | 0.027 | 681 | tags=37%, list=13%, signal=42% |
| 33 | ALMSTEDT\_VGR\_DIFF |  | 483 | -0.28 | -2.02 | 0.000 | 0.001 | 0.037 | 526 | tags=23%, list=10%, signal=23% |
| 34 | ANGIOGENESIS\_GO\_0001525 |  | 16 | -0.56 | -1.92 | 0.009 | 0.003 | 0.105 | 532 | tags=50%, list=10%, signal=55% |
| 35 | DOROSZKO\_U3180MG\_CLUSTER\_6 |  | 33 | -0.44 | -1.92 | 0.000 | 0.003 | 0.109 | 999 | tags=52%, list=19%, signal=63% |
| 36 | ROSEN\_KRONA\_TI\_UNPUBL |  | 45 | -0.39 | -1.85 | 0.005 | 0.006 | 0.201 | 394 | tags=22%, list=7%, signal=24% |
| 37 | KRONA\_ROSEN\_PERIVASCULAR\_INVASION |  | 47 | -0.38 | -1.79 | 0.010 | 0.009 | 0.306 | 421 | tags=23%, list=8%, signal=25% |
| 38 | KLING\_GBM\_PHENO\_PMID\_27667176 |  | 108 | -0.30 | -1.75 | 0.002 | 0.011 | 0.372 | 514 | tags=23%, list=10%, signal=25% |
| 39 | KRONA\_ROSEN\_WHITE\_MATTER\_INVASION |  | 180 | -0.27 | -1.70 | 0.002 | 0.016 | 0.484 | 519 | tags=19%, list=10%, signal=21% |
| 40 | NB\_KI\_CLUSTER23 |  | 153 | -0.28 | -1.67 | 0.000 | 0.019 | 0.552 | 981 | tags=34%, list=19%, signal=41% |
| 41 | DOROSZKO\_PERIVASCULAR\_INVASION |  | 33 | -0.37 | -1.58 | 0.031 | 0.030 | 0.730 | 319 | tags=30%, list=6%, signal=32% |
| 42 | RICHARDS\_NOWAKOWSKI\_SCIENCE\_2017\_GLYC\_UPREG |  | 410 | -0.22 | -1.55 | 0.000 | 0.037 | 0.816 | 1627 | tags=47%, list=31%, signal=62% |
| 43 | DOROSZKO\_U3013MG\_CLUSTER\_0 |  | 29 | -0.35 | -1.46 | 0.060 | 0.060 | 0.943 | 319 | tags=28%, list=6%, signal=29% |
| 44 | DOROSZKO\_U3013MG\_CLUSTER\_3 |  | 68 | -0.28 | -1.46 | 0.051 | 0.060 | 0.948 | 711 | tags=25%, list=13%, signal=29% |
| 45 | NB\_KI\_CLUSTER20 |  | 22 | -0.37 | -1.44 | 0.080 | 0.067 | 0.970 | 717 | tags=36%, list=14%, signal=42% |
| 46 | DOROSZKO\_U3054MG\_CLUSTER\_9 |  | 231 | -0.22 | -1.41 | 0.016 | 0.077 | 0.981 | 1314 | tags=32%, list=25%, signal=41% |
| 47 | GANDAL\_DEPRESSION\_DN\_PMID29439242 |  | 27 | -0.34 | -1.39 | 0.118 | 0.087 | 0.993 | 502 | tags=26%, list=9%, signal=28% |
| 48 | ALMSTEDT\_17Q\_MSIGDB |  | 177 | -0.21 | -1.30 | 0.063 | 0.138 | 1.000 | 708 | tags=18%, list=13%, signal=20% |
| 49 | GANDAL\_BIPOLAR\_UP\_PMID29439242 |  | 54 | -0.26 | -1.30 | 0.132 | 0.137 | 1.000 | 498 | tags=20%, list=9%, signal=22% |
| 50 | GANDAL\_SCHIZOPHRENIA\_UP\_PMID29439242 |  | 65 | -0.25 | -1.30 | 0.116 | 0.135 | 1.000 | 964 | tags=32%, list=18%, signal=39% |
| 51 | RICHARDS\_CAHOY\_JNEUROSCI\_2014\_MOG\_POS |  | 362 | -0.18 | -1.28 | 0.032 | 0.146 | 1.000 | 681 | tags=17%, list=13%, signal=19% |
| 52 | RICHARDS\_CAHOY\_JNEUROSCI\_2011\_ASTRO\_MATURE |  | 280 | -0.19 | -1.28 | 0.029 | 0.144 | 1.000 | 1048 | tags=26%, list=20%, signal=31% |
| 53 | DOROSZKO\_U3180MG\_CLUSTER\_5 |  | 23 | -0.30 | -1.17 | 0.279 | 0.255 | 1.000 | 391 | tags=26%, list=7%, signal=28% |
| 54 | LARSSON\_STATE\_5 |  | 73 | -0.22 | -1.15 | 0.259 | 0.274 | 1.000 | 1215 | tags=40%, list=23%, signal=51% |
| 55 | RICHARDS\_VERHAAK\_CANCERCELL\_2010\_NEURAL |  | 34 | -0.26 | -1.13 | 0.302 | 0.299 | 1.000 | 1603 | tags=44%, list=30%, signal=63% |
| 56 | DOROSZKO\_U3054MG\_CLUSTER\_10 |  | 148 | -0.19 | -1.13 | 0.236 | 0.295 | 1.000 | 343 | tags=12%, list=6%, signal=13% |
| 57 | NB\_KI\_CLUSTER15 |  | 91 | -0.20 | -1.11 | 0.292 | 0.317 | 1.000 | 1780 | tags=47%, list=34%, signal=70% |
| 58 | GANDAL\_ALCOHOLADDICTION\_UP\_PMID29439242 |  | 74 | -0.20 | -1.09 | 0.340 | 0.337 | 1.000 | 263 | tags=11%, list=5%, signal=11% |
| 59 | NB\_KI\_CLUSTER14 |  | 135 | -0.18 | -1.09 | 0.294 | 0.339 | 1.000 | 1490 | tags=41%, list=28%, signal=56% |
| 60 | GANDAL\_SCHIZOPHRENIA\_DN\_PMID29439242 |  | 21 | -0.29 | -1.09 | 0.354 | 0.336 | 1.000 | 502 | tags=24%, list=9%, signal=26% |
| 61 | ALLEN\_115\_OLIGOL4\_6MOBPCOL18A1 |  | 16 | -0.32 | -1.06 | 0.390 | 0.367 | 1.000 | 520 | tags=25%, list=10%, signal=28% |
| 62 | DOROSZKO\_U3054MG\_CLUSTER\_3 |  | 130 | -0.16 | -0.94 | 0.563 | 0.589 | 1.000 | 846 | tags=22%, list=16%, signal=25% |
| 63 | PARADA\_T2\_DN\_26461091 |  | 26 | -0.22 | -0.90 | 0.581 | 0.653 | 1.000 | 439 | tags=19%, list=8%, signal=21% |
| 64 | NB\_KI\_CLUSTER22 |  | 85 | -0.11 | -0.61 | 0.967 | 0.963 | 1.000 | 837 | tags=16%, list=16%, signal=19% |
Table: Gene sets enriched in phenotype **na **[plain text format]****
